# Comparing self reported and physiological sleep quality from consumer devices to depression and neurocognitive performance

**DOI:** 10.1038/s41746-025-01493-6

**Published:** 2025-02-09

**Authors:** Samir Akre, Zachary D. Cohen, Amelia Welborn, Tomislav D. Zbozinek, Brunilda Balliu, Michelle G. Craske, Alex A. T. Bui

**Affiliations:** 1 Medical Informatics Home Area, University of California, Los Angeles, 924 Westwood Blvd., Los Angeles, CA USA; 2 Department of Psychology, University of Arizona, 1503 E. University Blvd. (Building 68), Tucson, AZ USA; 3 Department of Psychiatry and Biobehavioral Sciences, University of California, Los Angeles, 760 Westwood Plaza, Los Angeles, CA USA; 4 Department of Pathology and Laboratory Medicine, Department of Computational Medicine, and Department of Biostatistics, University of California, Los Angeles, 621 Charles E. Young Dr. South, Los Angeles, CA USA

**Keywords:** Human behaviour, Machine learning, Sleep

## Abstract

This study examines the relationship between self-reported and physiologically measured sleep quality and their impact on neurocognitive performance in individuals with depression. Using data from 249 participants with medium to severe depression monitored over 13 weeks, sleep quality was assessed via retrospective self-report and physiological measures from consumer smartphones and smartwatches. Correlations between self-reported and physiological sleep measures were generally weak. Machine learning models revealed that self-reported sleep quality could detect all depression symptoms measured using the Patient Health Questionnaire-14, whereas physiological sleep measures detected “sleeping too much” and low libido. Notably, only self-reported sleep disturbances correlated significantly with neurocognitive performance, specifically with processing speed. Physiological sleep was able to detect changes in self-reported sleep, medication use, and sleep latency. These findings emphasize that self-reported and physiological sleep quality are not measuring the same construct, and both are important to monitor when studying sleep quality in relation to depression.

## Introduction

Sleep disturbance is a core symptom of depressive episodes, and sleep disorders commonly co-occur with major depressive disorder (MDD)^1^. While polysomnography is the gold standard for assessing sleep quality, it is challenging to use in naturalistic settings or over the extended periods of time typical of depressive episodes. Actigraphy from wrist-worn research-grade devices, which has been compared to polysomnography^2^, represents one alternative that is deployable in naturalistic settings. However, research actigraphy devices are not as easy to use or as prevalent as their consumer wearable device counterparts^3,4^. Researchers thus often rely on self-reported sleep quality, using tools like daily sleep diaries or retrospective questionnaires like the Pittsburgh Sleep Quality Index (PSQI), and find that changes in self-reported sleep quality are associated with changes in depression severity^5,6^.

Despite the common reliance on self-reported measures, there is low concordance between subjective assessments and physiological sleep measurements^7,8^, especially among individuals with higher depression severity^9^. Misalignments between self-reported and objective sleep data—referred to as misappraised poor sleep—are associated with worse neurocognitive functioning^10^, which is itself linked to increased depression severity^11^. This underscores the importance of understanding how physiological and subjective sleep quality measures relate to each other and to self-reported depression and neurocognitive outcomes.

Studies investigating both physiological and self-reported sleep quality often find that self-reported sleep quality is more strongly associated with depression and anxiety than physiological measures^12–15^. In contrast, Biddle et al. found that physiologically measured sleep efficiency (but not self-reported sleep efficiency) correlated with aspects of neurocognitive performance related to reaction time and executive functioning in elderly men with comorbid MDD and insomnia^16^. Moreover, Klumpp et al. found that self-reported and actigraphy-measured sleep quality are differentially related to brain activity during emotional processing tasks, suggesting they capture different aspects of sleep quality^17^. These findings highlight that subjective and objective sleep assessments may capture distinct constructs in relation to depression.

Previous studies have predominantly relied on single or two-night physiological sleep measurements or short-duration monitoring, extending up to nine nights, often in controlled environments with small sample sizes, limiting their generalizability to naturalistic, long-term settings^9,18^. A notable exception is a recent study by Zheng and colleagues, which found that in a sample of 6785 participants, Fitbit-measured variability of sleep duration and sleep onset time was associated with the risk of MDD^19^. However, the work by Zheng and colleagues and much of the existing literature has focused on overall depression or anxiety severity rather than individual symptoms, failing to capture the symptom-level nuances that are critical for personalized treatment approaches. Most studies also emphasize associations rather than evaluating the predictive power of sleep metrics for specific depression-related outcomes.

Advancements in consumer wearable technology offer a unique opportunity to overcome these limitations. Growing evidence supports the comparability of sleep-related metrics between medical-grade actigraphy and consumer wearable devices^20,21^, enabling larger sample sizes and extended monitoring periods in naturalistic settings. Consumer devices like the Apple Watch show promise for sleep monitoring but face accuracy challenges, particularly in detecting awake periods^21,22^. These gaps underscore the need for research investigating the complementary roles of physiological and self-reported sleep measures, particularly in their ability to predict individual depression symptoms. By leveraging extended monitoring with consumer-grade devices, this study aims to address these gaps and provide new insights into the predictive utility of subjective and objective sleep data for depression symptoms.

In the present report, we study the relationship between physiological and self-reported measures of sleep quality in populations with depression. The dataset used comes from 342 participants who were monitored using passive sensing and self-reported measures over 13 weeks as part of a larger trial investigating features of anhedonic depression (Wellcome Leap MCPsych). A subset of 249 was used in this analysis based on the high availability of sleep annotation data from the iPhone and Apple watch. From the passively sensed sleep annotation data, sleep quality features are extracted per night (sleep duration, bedrest duration, onset, efficiency, latency, etc.). These physiological sleep features were selected to align with commonly calculated features in actigraphy-based sleep studies^23^. Self-reported depression symptoms are taken from individual items in the Patient Health Questionnaire-14 (PHQ-14), and subjective sleep quality is assessed via the PSQI. We tested the hypothesis that self-reported and physiological sleep quality measure distinct constructs that are differentially associated with depression-related outcomes. Our analyses include: (1) correlations between corresponding self-reported and physiological sleep quality measures, (2) evaluating the utility of each modality for detecting individual depression symptoms, (3) examining their relationships to neurocognitive performance as an independent domain impacted by depression, and (4) investigating the capacity of physiological measures to predict changes in self-reported sleep quality over time. These analyses aim to provide insights into the complementary roles of self-reported and physiological sleep data in advancing sleep research in the context of depression.

## Results

The data used in this analysis are from the Operationalizing Digital PhenoTyping in the Measurement of Anhedonia (OPTIMA) study that collected data between October 2022 and April 2024. OPTIMA aims to measure behaviors related to anhedonia in the context of depression, relating observations to neural markers of anhedonia. This analysis uses data from 249 participants comparing their physiologically measured sleep quality to self-reported measures of sleep quality, depression, and TestMyBrain-based^24^ neurocognitive performance. Participant demographics outlined in Table 1.

**Table 1:** Participant demographics and treatment history

Demographics and treatment history	Yes	No
Sex–female	160 (64.5%)	88 (35.5%)
Family Income <100k	125 (50.4%)	123 (49.6%)
Non-Hispanic White	120 (48.4%)	128 (51.6%)
History of Psychotherapy	201 (81.0%)	47 (19.0%)
Depression diagnosis	172 (69.4%)	76 (30.6%)
Psychotherapy (in past 4 weeks)	112 (45.2%)	136 (54.8%)
Currently using medication for mental health	116 (46.8%)	132 (53.2%)
	**Mean**	**Std**
Age	33.78	11.86

There are 249 participants used in analysis. However, one participant is missing baseline assessments including demographic data.

In the study population, 83.5% (207) of 249 participants have moderate or severe depression at baseline, per PHQ-14 total score greater than or equal to 10. Note that the PHQ-14 is a self-report questionnaire adapted from the PHQ^25^ by Cohen, Cohen, & Fried (see Depression Symptom Response Project OSF site: https://osf.io/j6r3q/) that disentangles multi-symptom confounded items from the PHQ-9 (e.g., “Trouble falling or staying asleep, or sleeping too much” is split into two items: “Trouble falling or staying asleep” and “sleeping too much”) and adds two symptoms (libido and irritability).

### Correlation between physiological and self-reported sleep quality

**Fig. 1:**
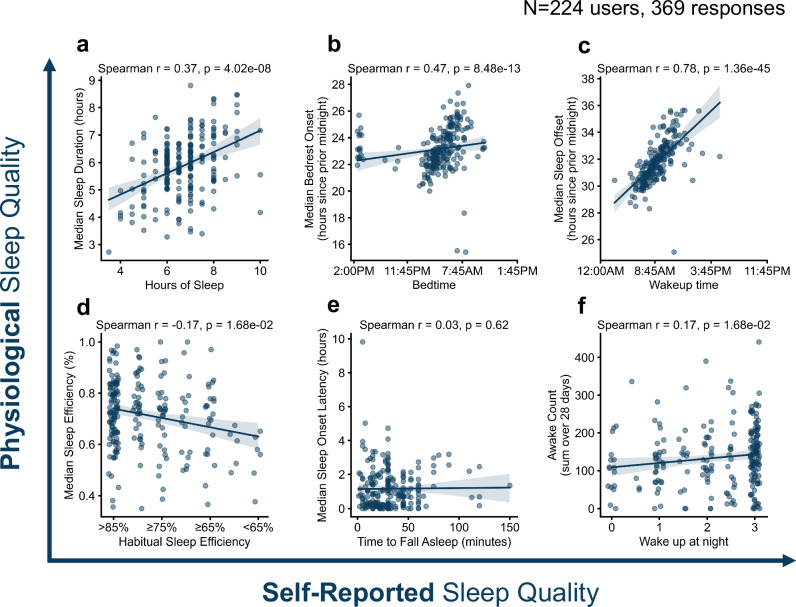
Correlation between physiological sleep parameters and self-reported sleep quality items or domains. *Y* axes represent physiological sleep parameters, and X-axes represent self-reported parameters. Comparisons are of (**a)** of median sleep duration to self-reported hours of sleep, **b** median bedrest onset to self-reported bedtime, **c** median sleep onset to self-reported wakeup time, **d** median sleep efficiency to self-reported habitual sleep efficiency, **e** median sleep onset latency to self-reported time to fall asleep, and (**f)** number of recorded night time awakenings to self-reported nightly awakenings where response is defined as: 0, not during the past month; 1, less than once a week; 2, once or twice a week; 3, three or more times a week.

Physiological sleep parameters measured over 28 days were correlated with six self-reported items or domains from the PSQI that had direct correspondences from 224 participants with 369 total responses. The mean of physiological (over 28 days) and self-reported (from two administrations of the PSQI) sleep quality was calculated. For example, in the case of the metric “median sleep duration,” the mean value of the “median sleep duration” from physiological data across the 28 days prior to each of the two PSQI assessments was compared to the mean of sleep duration from the two PSQI administrations. Of the six self-reported sleep quality items, five were significantly correlated with physiological sleep parameters (at False discovery rate (FDR) < 0.05) (Fig. 1a–d, f): sleep duration (Spearman’s *r* = 0.367, *p* = 4.02e-08), bedtime (Spearman’s *r* = 0.465, *p* = 0.47e-13), wakeup time (Spearman’s *r* = 0.781, *p* = 1.36e-45), habitual sleep efficiency (Spearman’s *r* = −0.166, *p* = 0.017), and nightly awakenings (Spearman’s *r* = 0.168, *p* = 0.017). Self-reported time to fall asleep showed a poor correlation with its physiological sleep counterpart (Spearman’s *r* = 0.033, *p* = 0.62; Fig. 1e). The correlation between self-reported and physiological sleep duration of 0.367 is slightly lower than Matthews et al. found in their full population (*r* = 0.40) but higher than they found in the highest quartile of depression symptoms (*r* = 0.23)^9^.

### Detecting depression symptoms with self-reported or physiological sleep quality

**Fig. 2:**
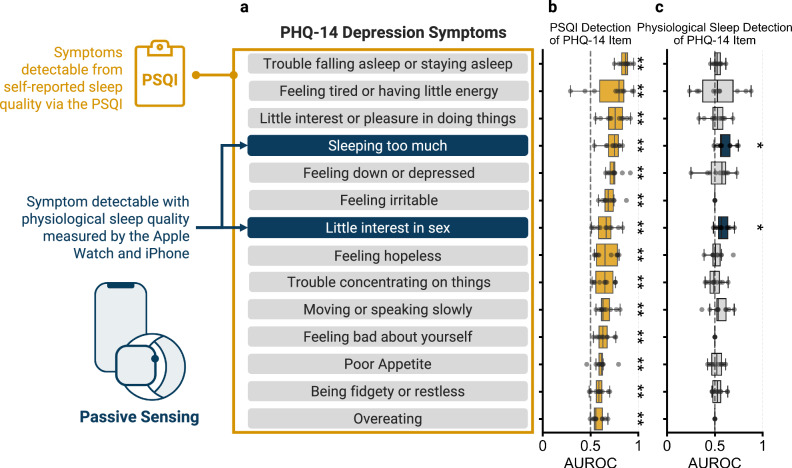
Machine learning model prediction of PHQ-14 items using physiological or self-reported sleep quality. **a** Symptoms of depression listed by order of relevance to self-reported sleep quality. Symptoms within the yellow box are detectable with self-reported sleep quality. Dark blue colored symptoms are detectable with physiological sleep data beyond random chance. **b** Performance of machine learning models to classify the presence or absence of PHQ-14 symptoms using item-level PSQI responses. **c** Performance of machine learning models to classify the presence of PHQ-14 symptoms using physiological sleep features. If AUROC is greater than 0.50 with a *p* value < 0.05 after correcting for FDR < 0.05 a star (*) or two stars if *p* < 0.01 (**) are annotated and the associated box is colored dark gold or dark blue.

Machine learning models were trained to detect symptoms of depression from the PHQ-14 (Fig. 2a) using either self-reported sleep quality (all individual items, domains, and total score) from the PSQI taken on the same day of the PHQ-14 (performance in Fig. 2b) or using physiological sleep parameters taken from the 8 days prior to PHQ-14 administration (performance in Fig. 2c). Models were trained to detect presence (or absence) of each PHQ-14 measured symptom (item response >0). 10-fold cross-validation was used to generate a distribution of model performance via area under the receiver operator curve (AUROC). In cross-validation, no data from individuals in the test set are present in the train set. The performance of models predicting PHQ-14 symptoms was tested to evaluate which symptoms can be detected at greater than random chance (AUROC > 0.5). Multiple comparisons were corrected using the Benjamini–Hochberg method with an FDR of 0.05. The design and validation of these models are described further in the Methods.

Of the 14 self-reported depression symptoms assessed on the PHQ-14, all were detectable above random chance using item-level responses to the PSQI. In contrast, only the symptoms “sleeping too much” (median AUROC = 0.5683, Wilcoxon Signed Rank test FDR-adjusted *p* = 0.0219) and “little interest in sex” (median AUROC = 0.5675, Wilcoxon Signed Rank test FDR-adjusted *p* = 0.0219) were detectable above random chance using physiological sleep quality. Model performance is further described in Table 2 and sensitivity and specificity of the best performing model are shown in Supplementary Table 2.

**Table 2:** Model performance for predicting PHQ-14 item response with either self-reported sleep quality or physiological sleep quality

	PSQI (*n* = 249 users, 705 responses)			Physiological Sleep (*n* = 247 users, 1565 responses)		
PHQ-14 item	Model	Median AUROC	Adjusted *P* value	Model	Median AUROC	Adjusted *P* value
Trouble falling asleep or staying asleep	RF	0.867	1.3E-03	GB	0.523	0.264
Feeling tired or having little energy	GB	0.798	0.010	LR	0.526	0.417
Little interest or pleasure in doing things	RF	0.758	1.3E-03	RF	0.530	0.417
Sleeping too much	LR	0.751	1.3E-03	RF	0.568	0.022
Feeling down, depressed	LR	0.743	1.3E-03	RF	0.574	0.344
Feeling irritable	LR	0.685	1.3E-03	D	0.500	1.000
Little interest in sex	LR	0.665	1.3E-03	LR	0.568	0.022
Feeling hopeless	LR	0.652	1.3E-03	GB	0.506	0.405
Trouble concentrating on things	RF	0.651	1.3E-03	LR	0.490	0.839
Moving or speaking slowly	LR	0.631	1.3E-03	GB	0.534	0.290
Feeling bad about yourself	LR	0.629	1.3E-03	D	0.500	1.000
Poor appetite	LR	0.616	2.3E-03	LR	0.521	0.402
Being fidgety or restless	LR	0.588	5.2E-03	GB	0.525	0.290
Overeating	LR	0.556	2.3E-03	D	0.500	1.000

*LR* logistic regression, *RF* random forest, *GB* gradient boosting, *D* Dummy. underline = FDR adjust *p* value < 0.05 for model AUROC > 0.5.

**Fig. 3:**
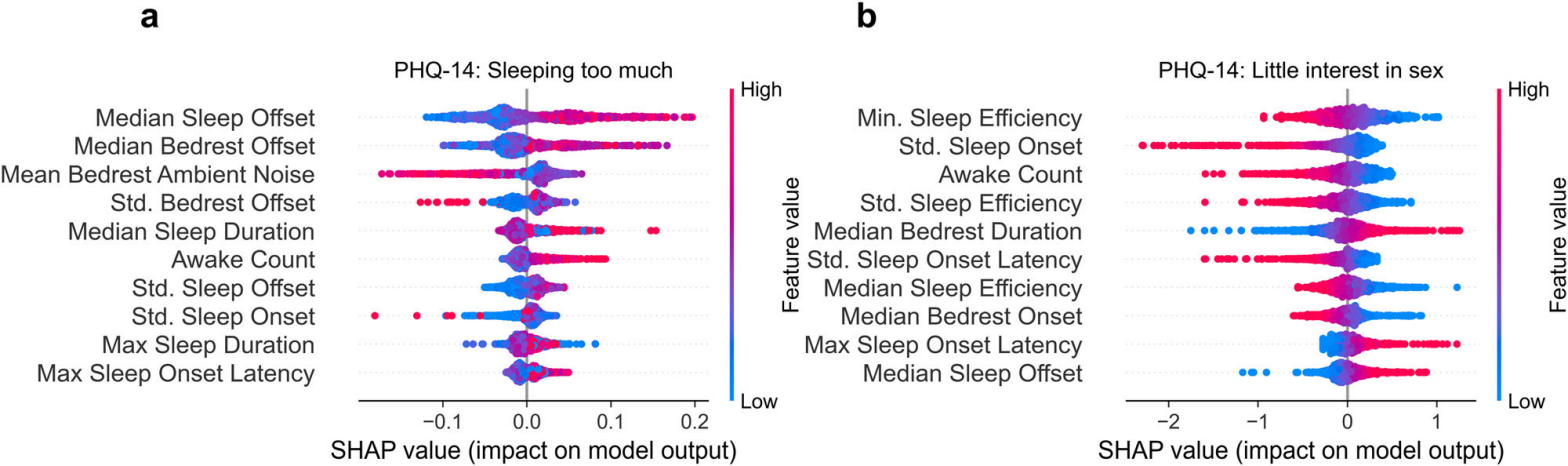
Feature importance for machine learning model prediction of PHQ-14 items using physiological sleep features. Feature importance via SHAP value for models predicting PHQ-14 items “Sleeping too much” and “Little interest in sex”. Each dot represents a prediction within the test set of a cross-validation fold. Colour indicates relative feature value (e.g., higher mean heart rate is red, lower mean heart rate is blue). SHAP value indicates how influential a feature was to a single prediction. The sign (positive or negative) of the SHAP value denotes whether the importance was for classifying as a negative or positive class (low vs high class). The magnitude of the SHAP value indicates its importance to the model for a prediction.

For models using physiological sleep whose AUROC is significantly greater than 0.5 (FDR < 0.05), feature importance is examined to investigate which features appear important to model performance and how they relate to model decision-making. Feature importance was analyzed using SHapley Additive exPlanation (SHAP) scores^26^. SHAP feature importance scores help explain each individual prediction from a model, allowing researchers to understand how different feature values impact model decisions in the testing set. Higher sleep offset, bedrest offset times with longer bedrest durations, and quieter bedtime environments are used by the model to detect “sleeping too much.” While maximum sleep duration is also a highly ranked feature by the model, its association with self-reported sleeping too much does not appear linear (i.e., higher max sleep duration is not consistently used by models to detect sleeping too much; Fig. 3a). The model detecting “little interest in sex” finds low sleep efficiency, less variable sleep onset, and longer time in bed as associated with the depression symptom (Fig. 3b).

For models using physiological sleep to detect PHQ-14 item responses that had performance greater than random chance, performance was compared across baseline depression severity (PHQ-14 total score ≥10), baseline anhedonia (PVSS total score <5), family income (≥100k USD), race (non-Hispanic white vs. all), and sex at birth. No significant differences in AUROC were found in performance between groups.

### Correlations between neurocognitive performance and i) physiological sleep quality and ii) self-reported sleep quality

**Fig. 4:**
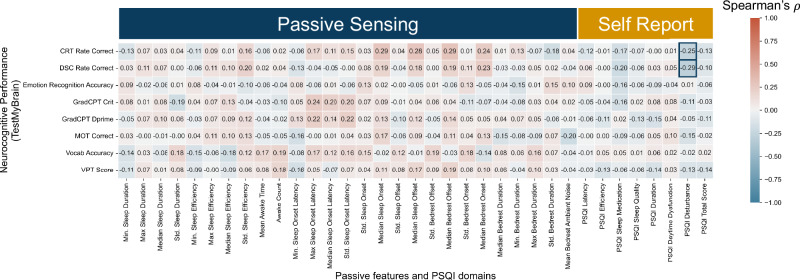
Correlations between sleep quality and neurocognitive performance. Spearman correlation of watch-derived median features over a 28-day period and PSQI domains and total score with TestMyBrain neurocognitive performance metrics. Correlations with a *p* value < 0.05 after multiple testing corrections (FDR < 0.05) have a box drawn around them.

Neurocognitive performance was measured via TestMyBrain: a digital neuropsychological battery of cognitive tests, including working memory, sustained attention, and inhibition^24^. Measures from TestMyBrain were correlated with self-reported sleep quality domains from the PSQI taken on the same day and physiological sleep parameters aggregated over the prior 8 days (Fig. 4). Only the sleep disturbances domain was found to correlate with neurocognitive performance measures of processing speed from the Digit Symbol Coding (DSC) test rate correct score (Spearman’s *r* = −0.29, FDR-adjusted *p* value = 0.009) and the choice reaction time (CRT) test rate correct score (Spearman’s *r* = −0.25, FDR-adjusted *p* value = 0.037).

In contrast to Gualtieri et al.^11^ we do not find a significant correlation between depression severity (PHQ-14 total score) and neurocognitive performance (FDR-adjusted *p* values all >0.05). This finding may in part be driven by few participants with low depression severity in the OPTIMA study.

### Detecting changes in self-reported sleep quality using physiological sleep data

Several domains of self-reported sleep quality can be detected using physiological sleep alone. These self-reported domains are daytime dysfunction due to sleepiness (median AUROC = 0.602, FDR-adjusted *p* = 0.008), sleep disturbances (median AUROC = 0.589, FDR-adjusted *p* = 0.049), and sleep duration (median AUROC = 0.632, FDR-adjusted *p* = 0.012).

**Fig. 5:**
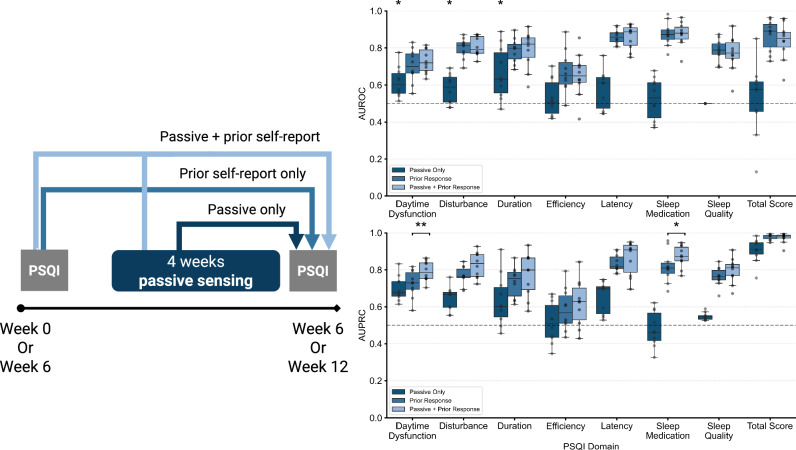
Performance of predictive models at detecting domains and total scores of the Pittsburgh Sleep Quality Index (PSQI) with different input feature sets. Top plot shows AUROC score value; if *p* value for passive-only detection greater than random chance is < 0.05 after controlling FDR < 0.05, a star is annotated. Bottom plot shows average precision performance; if passive data with prior response improves performance over just prior response, with a *p* < 0.05, it is annotated with a star (*) or two stars if *p* < 0.01 (**).

To assess if physiological sleep quality can detect changes to self-reported sleep quality, three models were trained using i) only physiological sleep quality, ii) physiological sleep quality and 6-weeks prior self-report answers, and iii) only prior self-report answers. For two domains (daytime dysfunction due to sleepiness and sleep medication), performance improved when adding physiological data (Fig. 5), with median AUPRC difference with and without passive data = 0.046 (FDR-Adjusted *p* = 0.008) and 0.064 (FDR-Adjusted *p* = 0.039), respectively, indicating that physiological data can help models better detect the positive class without sacrificing overall performance. No significant difference was observed based on performance measured via AUROC.

## Discussion

In Fig. 1, we demonstrate that while physiological sleep measures are related to their corresponding self-reported PSQI items, they often assess distinct constructs. For example, physiological metrics capture objective data, whereas PSQI items reflect subjective perceptions. As a validation of consumer wearable-based sleep assessment, we confirm the expected correlation between physiological and self-reported sleep duration, consistent with prior findings^9^ and observed in the OPTIMA dataset. However, for measures such as nightly awakenings, sleep onset latency, and sleep efficiency, we observe low correlations between physiological and self-reported data, with Pearson correlation coefficients below 0.2. This disparity reflects the fundamental difference between perceived and objectively measured sleep metrics.

Our findings also highlight notable potential limitations of physiological sleep measurements using consumer devices. For instance, it is highly unlikely that any participant would average zero nightly awakenings over a 30-day period. Yet, in Fig. 1f, nine participants were recorded with zero detected awakenings, illustrating the limited sensitivity of actigraphy-based sleep monitoring in detecting awake periods^21^. Additionally, we noted instances of overestimation in sleep onset latency, including one outlier reporting nearly 10 hours to fall asleep. This tendency to overestimate onset latency aligns with prior reviews, such as that by Scott and colleagues^27^. These observations underscore the need to interpret physiological sleep metrics from consumer devices cautiously, particularly when examining nuanced aspects of sleep quality.

The finding that all 14 symptoms of depression from the PHQ-14 are detectable using self-reported sleep quality highlights the interconnection amongst different self-reported measures related to depression. While sleep quality-related items are part of the PHQ-14 (e.g., sleeping too much), many other items, such as “little interest or pleasure in doing things” are not directly related to sleep and yet are detectable from self-reported sleep quality with good performance (AUROC values above 0.75). In comparison, physiological sleep was only able to detect the PHQ items of “sleeping too much” and “little interest in sex.” This observation is in line with prior work suggesting that physiological sleep is not as strongly associated with depression symptomology as self-reported sleep quality^12–15^. Additionally, physiological sleep appears to be an insufficient detector for depressive symptoms that may require other social behavior features measurable from phones and watches, as illustrated in prior work^28–34^. However, by focusing on individual self-report items using the PHQ-14 instead of overall depression severity, we highlight that for specific symptoms (e.g., sleeping too much and low libido), physiological sleep may provide meaningful insights in depressed populations.

The differences in predictive performance between self-reported and physiological sleep measures likely reflect the nature of the data collected by each method. Self-reported measures inherently capture subjective experiences, which may integrate broader psychological and emotional factors relevant to depressive symptoms. In contrast, physiological data represent objective sleep patterns and are constrained by what can be directly and reliably measured. This distinction underscores the role of the administration method in shaping the relationship between subjective and objective sleep measures. For example, endorsement of “little interest in sex” was associated with low sleep efficiency but high bed rest duration. Prior studies have found associations between self-reported sleep duration and libido^35,36^, but the modeling work herein suggests that in cohorts with depression, the relationship between libido and sleep quality may be driven more by sleep efficiency and bedrest duration rather than total sleep duration.

The detection of “sleeping too much” as opposed to “trouble falling or staying asleep” also shows the benefit of separating traditionally combined symptoms in self-report scales. “Trouble falling or staying asleep” may be associated with rumination or bouts of wakefulness during sleep, whereas “sleeping too much” is more likely to be reflected in later sleep and bedrest offset times that can be objectively measured, a finding reflected in the top features used by the model to detect “sleeping too much.”

In relation to Neurocognitive performance, work by Zavecz and colleagues showed no relationship between overall subjective sleep quality and working memory^37^. Similarly, we did not find any significant correlations between either overall subjective sleep quality (PSQI total score) or physiological measures of sleep duration, efficiency, latency, and neurocognitive performance. It is important to note, however, that the absence of significant correlations does not imply the absence of a relationship, as such findings could be influenced by limited statistical power, variability in the sample, or potential measurement constraints^38^. Despite this, the observed lack of correlations highlights the complexity of the relationship between sleep and neurocognitive performance, which may also be influenced by unmeasured factors such as baseline cognitive ability. Future studies with larger and more diverse samples may be necessary to clarify these relationships. Notably, we did find that higher levels of self-reported sleep disturbances correlated with decreasing processing speed, underscoring the potential value of subjective sleep measures for capturing neurocognitive impacts.

In traditional mental healthcare, patient interactions with clinical professionals or the completion of self-reports occur infrequently. By employing continuous passive monitoring, we can detect changes in self-reported sleep medication use and sleep latency, allowing for the identification of significant variations in these areas. These findings illustrate how integrating self-reported and objectively measured sleep quality parameters can enhance patient care.

In the analysis presented in Fig. 5, increases in model performance as measured by AUPRC occur when adding passive sensing data to self-reported sleep quality, although the same does not occur for AUROC. An improvement in AUPRC without a significant change in AUROC suggests that the model improvement is centered on better identifying the positive class. For example, it can be important to detect endorsement of sleep medication use, as it serves as an objective index of having experienced sleep problems; incorporating passive sensing data can significantly enhance the detection of sleep medication usage.

Additionally, we investigated whether variability in user responses contributed to differences in predictive performance by analyzing the median absolute difference per PSQI domain and the percentage of users who changed their binarized response between assessments. As shown in Supplementary Fig. 4, the two domains where the addition of passive data improved detection—daytime dysfunction due to sleepiness and sleep medication use—represent opposite extremes of variability, with daytime dysfunction showing the highest variability (29.9% of users changed response) and sleep medication use showing the lowest (16.0% of users changed response). These findings suggest that response variability is not the primary driver of differences in predictive performance, further highlighting the potential of passive sensing data to complement self-reported measures.

The data reported here suggests that physiological sleep from consumer devices could augment how self-reported sleep quality measures are interpreted and enable continuous passive sensing of sleep quality-relevant symptomology over time. Ultimately, passive data could be used to recommend users with specific digital phenotype profiles to seek a mental healthcare professional for further evaluation (e.g., to complete self-report questionnaires, to obtain diagnostic evaluation, or to receive treatment). This approach could greatly facilitate the user’s awareness of their own functioning, increase the number of people who receive treatment, and reduce the delay between symptom onset and treatment initiation. The data could also be used by the user and healthcare professional to monitor treatment progress and mechanisms, as well as inform when treatment termination is advised or if lapses/relapses occur.

However, it is important to acknowledge that the performance of models using only physiological sleep data to predict PHQ-14 items remains modest, with AUROC values generally just above 0.5 and below 0.6. This performance indicates that while physiological sleep metrics may offer valuable context for understanding an individual’s sleep patterns, they are not yet sufficiently reliable to serve as standalone indicators for clinically relevant symptoms. These findings underscore the current limitations of physiological sleep data in detecting mental health symptomatology and highlight the need for integration with other data sources, such as self-reported measures or additional physiological indicators, to improve predictive accuracy. As the technology and modeling approaches evolve, these limitations could be mitigated, but at present, self-reported measures remain essential for clinical assessment and decision-making.

Sleep stage annotation (rapid-eye movement, deep, etc.) data is available for participants, but across the duration of the study, it is uncertain how changes in operating system versions, and updates to annotation algorithms from Apple HealthKit may have influenced comparability of these annotations. For that reason, this work centers on measures such as sleep efficiency, duration, latency, and others that only rely on differentiation of sleep vs. bed rest. Similarly, of the 342 participants in the study, only 249 have any sleep annotation data available from Apple HealthKit, limiting the sample size used in this work. This missing data was caused by a dependency on setting up approximate bedtime and intended wakeup times within iOS, not known at the onset of the study, but included within onboarding instructions once discovered.

It is important to contextualize these results in the population represented by the parent study, which recruited participants with moderate to severe depression and across the full spectrum of anhedonia severity, resulting in a symptomatically distinct population with the goal of understanding anhedonic depression. The models trained and tested here will have different performances on a sample more representative of the American population or even a population of participants with depression. Additionally, there is a heavy skew in socioeconomic characteristics of this population, with 50.4% of participants having annual family incomes >$100 K USD.

As shown in Table 3, several PHQ-14 items like “feeling tired or having little energy” have class imbalances (97% positive class) when converted to binary outcomes in this study population. This imbalance likely contributes to being unable to predict presence or absence of the symptom greater than random chance. One potential remedy for future analyses would be useful to identify what a meaningful difference in item response is per participant.

**Table 3:** Distribution of self-reported survey responses within OPTIMA study, including baseline assessments

Survey	Question	Mean	Std	Threshold	% True	# Responses
PHQ-14	Total score	12.78	4.92	10	73%	2162
	Little interest or pleasure in doing things	1.53	0.84	1	91%	2162
	Trouble concentrating on things	1.66	0.96	1	87%	2162
	Moving or speaking slowly	0.34	0.69	1	24%	2162
	Being fidgety or restless	0.83	0.96	1	52%	2162
	Feeling irritable	1.5	0.9	1	88%	2162
	Little interest in sex	1.6	1.12	1	78%	2080
	Feeling down, depressed	1.57	0.86	1	92%	2162
	Feeling hopeless	1.19	0.95	1	74%	2162
	Trouble falling asleep or staying asleep	1.64	1.06	1	83%	2162
	Sleeping too much	0.96	1.03	1	56%	2162
	Feeling tired or having little energy	2.13	0.86	1	97%	2162
	Poor appetite	0.87	0.95	1	56%	2162
	Overeating	1.01	1.01	1	60%	2162
	Feeling bad about yourself	1.51	1	1	83%	2162
PSQI	Total score	8.89	3.46	5	90%	705
	Daytime dysfunction	1.7	0.71	2	61%	705
	Disturbance	1.58	0.63	2	54%	705
	Duration	0.7	0.85	1	50%	705
	Efficiency	0.69	0.97	1	41%	705
	Latency	1.67	1.02	2	57%	705
	Sleep medication	0.9	1.22	1	41%	705
	Sleep quality	1.64	0.72	2	57%	705

Causality and temporality were not explored in this investigation of sleep qualities associated with depression. Future analyses could benefit from employing advanced statistical methods, such as Dynamic Structural Equation Modeling, to examine the bidirectional relationships between sleep parameters and depression symptoms. Such approaches could provide deeper insights into the temporal dynamics and causal interactions that static analyses cannot capture. Additionally, the sample size of 249 participants limits the feasibility of applying more complex machine learning or deep learning models, as these would require substantially larger datasets to train effectively without overfitting.

The findings of this study highlight key differences in the utility of physiological and self-reported sleep measures for understanding depression and related neurocognitive performance. While self-reported sleep quality demonstrated strong predictive relationships with all PHQ-14 depression symptoms, physiological sleep measures were only able to detect two symptoms: “sleeping too much” and “little interest in sex.” This contrast reflects the complementary nature of these methods: self-reports capture subjective perceptions that integrate broader psychological and emotional dimensions, whereas physiological measures provide objective data constrained by what is measurable through consumer devices. Furthermore, the weak correlations between physiological and self-reported sleep measures underscore that these methods do not measure the same construct and are not interchangeable. Neurocognitive performance, which was significantly associated with self-reported sleep disturbances but not with physiological sleep measures, further emphasizes the distinct contributions of these modalities. Taken together, these findings suggest that while physiological sleep data from consumer devices can augment self-reports by offering objective insights, they currently lack the sensitivity and breadth needed to replace self-reported measures. Future research should explore integrative approaches that leverage both modalities to more comprehensively assess the interplay between sleep and depression.

## Methods

### Dataset and study description

The parent study recruited participants with medium or high depression severity and low, medium, or high levels of anhedonia. The participants were not treatment-seeking, but the recruitment process ensured that the population represented the clinical characteristics relevant to depression and anhedonia.

As part of the study, there is extensive digital phenotyping data collected from participants using their own iPhones and a study-provided Apple Watch Series 7 or higher over the course of 13 weeks. Participant characteristics of those enrolled in the study are shown in Table 1. The UCLA Depression Grand Challenge Study App (DGC Study App) built by Avicenna Research is installed on participant iPhones and used to collect digital health data. The DGC Study App uses HealthKit and SensorKit APIs for passive measures and deploys ecological momentary assessments (EMAs).

All research study activities were reviewed and approved by the UCLA Neuroscience institutional review board MIRB3 (IRB00004473). All research study participants signed informed consent for the study protocol (#22-000059) prior to participation.

### Self-report measures

For this analysis, responses from two self-report questionnaires are investigated to compare digital health sensor data to depression and sleep quality.

1. A modified version of the Patient Health Questionnaire Depression Scale 9 (PHQ-9) is used that has 14 total items, referred to as the PHQ-14. The PHQ-9 has high internal reliability (Cronbach’s alpha = 0.89)^25^. Modifications include splitting compound symptoms (i.e., appetite decrease vs. overeating, sleep increase vs. decrease, psychomotor agitation vs. retardation, feeling down or depressed vs. feeling hopeless), adding two items to assess irritability and libido (i.e., “little interest in sex”), and removing the suicidality item. A recent individual participant data meta-analysis demonstrated the equivalence of the PHQ-8 and PHQ-9 for screening/diagnosis^39^. For this study, a total score representing the PHQ-8 was created by taking the max score of each pair of separated compound symptoms and excluding the two added items, where higher scores indicate greater depression. The PHQ-14 is administered 9 total times at Weeks 0, 1, 2, 4, 6, 7, 8, 10, and 12.
2. The PSQI asks participants to rate their prior 1-month of sleep and assesses sleep quality and disturbances^40^. The PSQI asks 19 questions which are used to calculate 7 subscales: subjective sleep quality, sleep latency, sleep duration, habitual sleep efficiency, sleep disturbances, use of sleeping medication, and daytime dysfunction. The subscales are added to form a global score, where higher scores indicate worse sleep. The 7 subscales and total score are used as detection targets. The PSQI has a high internal reliability (Cronbach’s alpha = 0.8)^41^. The PSQI is completed at Weeks 0, 6, and 12.

For the PHQ-14 there are two versions used across the study, one which asks participants to rate their past single week (7/9 administrations), and another that asks about the past two weeks (2/9 administrations). For all questionnaires, item-level responses, subscales (also referred to as domains), and total scores are converted to binary outcomes enabling binary classification machine learning models to be trained. For the PHQ-14, questions are binarized to endorsing or not endorsing a symptom, and the total score is converted to binary based on a score ≥10 to match depression screening guidelines^42^. The PSQI domains and total scores are converted to binary as follows:

- Daytime dysfunction: requires a cause of daytime dysfunction weekly.
- Disturbances: requires at least 3 causes of sleep disturbance ≥3 times a week.
- Duration**:** requires <7 hours of sleep on average. Picked because the recommendation for adults are 7–9 hours.
- Efficiency (HSE): requires sleep efficiency <85%. Picked because recommendations for adults are >80%.
- Latency**:** requires ≥30 minutes to fall asleep less than once a week AND average time to sleep ≥15 minutes.
- Sleep medication: any vs no sleep medication
- Sleep quality: good vs bad distinction (scores of 3 and 2 correspond to very bad and fairly bad)
- PSQI total score ≥5 is used for sleep disorder screening^40^.

Class balance for PHQ-14 and PSQI items is shown in Table 3.

### TestMyBrain measures

TestMyBrain includes a set of standardized computer-based tasks used to assess neurocognitive performance in several domains^24,43^. OPTIMA’s protocol utilizes a subset of these assessments, the DSC test, CRT test, multiple object tracking (MOT) test, emotional recognition test (ERT), vocabulary accuracy test (VAT), gradual onset continuous performance test (GradCPT), and verbal paired association (VPT) test. From each of these tests the output metrics suggested for use by TestMyBrain are calculated:

1. Accuracy (for VAT and ERT) is the proportion of correct responses per test in a trial
2. Rate correct score (for DSC and CRT) is a combined metric of speed and accuracy. It is calculated as shown in Eq. (1).
  1 $${Rate\; Correct\; Score}=1000* \frac{{Accuracy}}{{median\; Reaction\; Time}}$$
3. D-prime (${d}^{^{\prime} }$; for GradCPT) is a measure of the user’s sensitivity and is reported from TestMyBrain
4. Crit (for GradCPT) is a measure of user bias reported from TestMyBrain
5. Correct (for MOT) is the proportion of correctly identified targets during the task

### Physiological sleep feature generation

Digital health sensor features were generated by aggregating sensor data prior to self-report administration. Input features are aggregated based on all data available within a date range which ends at the timestamp of self-report administration and begins N-days prior. For the PSQI, participants are asked about their last month, so 28 days of digital health sensor data prior to the timestamp of administration are collected per participant response. For the PHQ-14, participants are asked about 7 or 14 days prior to the timestamp of administration; however, to make input features comparable, 8 days of sensor data are aggregated prior to assessment, both when participants are asked about the last week or 2 weeks. A time span of 8 days has been shown by Sun et al. to be a useful minimum span when using smartphone data to predict the PHQ-8^44^. All sensor data is collected utilizing Apple’s HealthKit application programming interface (API). Note that measures about time in bed are reported in Apple HealthKit as being derived from the iPhone, but actual sleep annotations (e.g., asleep or detailed sleep staging) are exclusively obtained from the Apple Watch and are based on wrist-based actigraphy. A total of 27 physiological sleep features are generated. Distributions of features after aggregation prior to PHQ-14 and PSQI assessments in the supplementary materials.

Annotations of bedtime and sleep times from Apple Health annotations are used to calculate bedrest duration (time in bed), sleep duration, sleep efficiency, sleep onset latency, and night awake time each day between 3 pm the day prior to 3 pm the day of metric reporting. These values are aggregated over the 28- or 8-day period prior to PSQI or PHQ-14 administration. Sleep duration, bedrest duration, sleep efficiency, and sleep onset latency are aggregated by taking the minimum, maximum, median, and standard deviation per day. Nightly awakenings are aggregated as the mean hours and count of awakenings. Sleep onset, sleep offset, bedrest onset, and bedrest offset are aggregated by taking the median and standard deviation. Noise during sleep is aggregated as the mean noise during bedrest periods over the aggregation time window.

Watch wear hours are determined by the number of hours that participants have at least one heart rate log. The percentage of watch wear hours is calculated for the relevant span of data prior to a given self-report. Watch wear is not included as a predictive feature but rather used for quality control. Self-reports with less than 80% of watch wear hours during the relevant timespan prior to assessment are removed from analysis. If participants did not set their sleep schedules via the phone operating system, automatic sleep detection would not occur and there would be no sleep annotations for a participant even if the watch was worn during sleep. For the purposes of this analysis which centers on sleep quality measurements, records without sleep annotation data are removed. If the sleep schedule was set, sleep annotation data were still generated for periods outside of the scheduled sleep window. However, these periods lacked detailed sleep staging information (e.g., REM, deep sleep) and were simply marked as “asleep.” Sleep staging was not used for features generated in this work. Missing data is described in Table 4.

**Table 4:** Availability of physiological sleep parameters aggregated prior to self-report administration of the PSQI and PHQ-14

	PSQI (28-day)			PHQ-14 (8-day)		
Passive sensing feature	Count	Missing	% Missing	Count	Missing	% Missing
Min. sleep duration	364	0	0%	1565	0	0%
Max sleep duration	364	0	0%	1565	0	0%
Median sleep duration	364	0	0%	1565	0	0%
Std. sleep duration	355	9	3%	1503	62	4%
Min. sleep efficiency	364	0	0%	1565	0	0%
Max sleep efficiency	364	0	0%	1565	0	0%
Median sleep efficiency	364	0	0%	1565	0	0%
Std. sleep efficiency	355	9	3%	1503	62	4%
Mean awake time	364	0	0%	1565	0	0%
Awake count	364	0	0%	1565	0	0%
Min. sleep onset latency	364	0	0%	1565	0	0%
Max sleep onset latency	364	0	0%	1565	0	0%
Median sleep onset latency	364	0	0%	1565	0	0%
Std. sleep onset latency	355	9	3%	1503	62	4%
Std. sleep onset	355	9	3%	1503	62	4%
Median sleep onset	364	0	0%	1565	0	0%
Std. sleep offset	355	9	3%	1503	62	4%
Median sleep offset	364	0	0%	1565	0	0%
Std. bedrest offset	362	2	1%	1551	14	1%
Median bedrest offset	364	0	0%	1565	0	0%
Std. bedrest onset	362	2	1%	1551	14	1%
Median bedrest onset	364	0	0%	1565	0	0%
Median bedrest duration	364	0	0%	1565	0	0%
Min. bedrest duration	364	0	0%	1565	0	0%
Max bedrest duration	364	0	0%	1565	0	0%
Std. bedrest duration	362	2	1%	1551	14	1%
Mean bedrest ambient noise	301	63	21%	1298	267	21%

### Correlation analysis

To compare watch-derived sleep and self-reported sleep with neurocognitive performance, watch features were aggregated from 28 days prior starting from the timestamp of the PSQI administration taken the day participants performed the TestMyBrain assessment. In this way, features from the watch represent the same timespan that the self-report is intended to measure. Each watch feature and PSQI domain is correlated with each of the TestMyBrain performance measures using a Spearman correlation and p-values are adjusted using the Benjamini–Hochberg method, controlling the FDR correction (*α* = 0.05).

### Machine learning pipeline

**Fig. 6:**
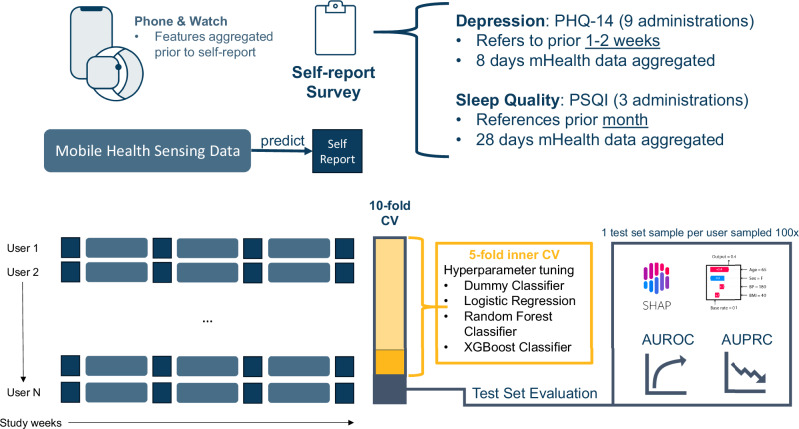
Machine learning pipeline for using physiological sleep data to detect self-report items and summary scores. Diagram depicts the use of nomothetic modeling approach with a 10-fold cross-validation used to assess model performance.

Classification models are trained to use the passive sensor features, prior survey response, or both to classify survey items and total score responses; an overview of the machine learning methods is shown in Fig. 6. All sensor data collected eight days (for PHQ-14) or 28 days (for PSQI) prior to the timestamp of a self-report administration is used to classify self-report response. Although the PHQ-14 asks participants about either their prior week or 2 weeks, eight days of aggregation is chosen based on findings from Sun et al. predicting PHQ-8^44^. Models are trained in a way that splits participants used in training from those used to evaluate a model. Given how uniquely identifiable individuals are from their vitals taken by mobile health devices^45^, if the participant level split is not done, models may simply learn to predict a participant training set score.

Of the 342 OPTIMA participants who completed the study, 249 have sleep annotation data from the Apple Watch and are included in this analysis. All 249 participants with any sleep annotation data have PHQ responses totaling 1850 assessments (excluding baseline assessment). After quality control for watch wear covering at least 80% of hours for 8 days prior, 1565 PHQ-14 responses from 247 participants are used in the analysis. There are 242 participants with PSQI responses totaling 457 PSQI assessments. After quality control (80% of hours for 28 days prior) there are 365 PSQI responses from 221 participants were used in the analysis. The above numbers do not include baseline or intake assessments as they were at the beginning of the study and do not have associated physiological sleep data. Participants have up to 8 responses to the PHQ-14 with associated sensor data, and up to 2 responses to the PSQI. When using PSQI item-level responses to predict PHQ-14 symptoms, baseline assessments are included allowing for 705 responses from 249 participants used in the machine learning modeling.

The 249-participant subset was selected for the presence of sleep annotation data; other missing data elements in the physiological sleep data are filled in using median imputation within cross-validation folds as described below. No imputation is done of self-reported measures.

For each classification task, k-fold nested cross-validation was done to select the best model and hyperparameter combination using a random search cross-validation approach. Internal cross-validation folds were created using a standard fivefold cross-validation. The outer fold was a stratified group 10-fold cross-validation keeping participants split across train and test sets. The pipeline for model training comprised median imputation, variance thresholding (features must have >0 variance in train set), feature selection, and robust scaling (5 to 95th percentile) before features reached the classification model. All implementation comes from standard functions in scikit-learn 1.2.2^46^.

Models used were the gradient boosting classifier (XGBoost), random forest (RF) classifier, logistic regression, and a dummy classifier (predicts mean of train data responses) to act as a baseline. For RF and XGBoost classifiers, hyperparameters were the number of estimators (100, 200, or 500), max depth (5, 10, or 20), minimum samples per leaf (2, 5, 10), max features (none, square root of total, log base 2 of total). All models other than the dummy classifier also had parameters for select-K-best feature selection. These were a number of features (10 or all) and the feature selection scoring function (mutual information-based or F-score based).

### Statistical analysis

The metrics of the area under the precision-recall curve (known as AUPRC or average precision) and receiver operator characteristic AUROC are calculated for each fold of cross-validation. AUROC is used as the primary performance metric, with AUPRC used to distinguish model performance where there may be a heavy class imbalance. To account for the differing number of repeated measurements in the test set per individual, one test set sample per individual is drawn 100 times per fold. For each test set fold AUROC and AUPRC are calculated.

To confirm if models using passive data only are performing greater than by random chance, a 1-sided Wilcoxon signed rank test was conducted on the AUROC value across the 10-folds to determine if the median AUROC was >0.5. As we are investigating the performance of 22 models (one per item response, domain score, or total score), Benjamini–Hochberg method is applied per survey to control for the FDR (*α* = 0.05), and models with a p-value less than 0.05 after correction are examined further. For the PSQI to determine if passive data can enable detection of changed response with data on a participant’s prior response, a paired *t* test comparing if models with the feature “prior response only” perform worse than “prior response with passive data” was performed for each PSQI domain and total score on both AUPRC and AUROC. Multiple testing correction is applied to results with an FDR of 0.05. The performance of models with the addition of passive data was considered significantly better than the prior response only if the adjusted *p* value was <0.05.

The performance for items and subscales where models had statistically significant aggregate performance is examined for discrepancies in performance across subgroups. Subgroup evaluation is done across baseline depression severity (PHQ-14 total score ≥10) and anhedonia (PVSS total score < 5), family income (≥ 100k USD), race (non-Hispanic white vs all), and sex at birth. No correction is done for multiple testing to investigate differences in performance across subgroups. To assess performance differences across groups, each test set fold is separated based on participants belonging to one of the groups. To account for repeated samples per participant, 1 sample is taken per participant 100 times before calculating AUROC per fold. Mann–Whitney *U* test is performed to determine if median AUROC is different across groups. All statistical testing is done using the Pingouin package version 0.5.5^47^ in Python version 3.11.6.

## Supplementary information

Supplementary Materials

## Data Availability

The datasets generated and analyzed during the current study are available from the corresponding author upon reasonable request.

## Code availability

The code that supports the findings of this study is available online at https://github.com/akre96/OPTIMA_sleep_quality.
