# Supplementary material for: Comparing self reported and physiological sleep quality from consumer devices to depression and neurocognitive performance
Source: NPJ Digit Med. 2025 Feb 9;8:92. doi: 10.1038/s41746-025-01493-6 (PMC11808080; doi:10.1038/s41746-025-01493-6)
Supplement: Supplementary file 1 — Supplementary Materials [file 41746_2025_1493_MOESM1_ESM.pdf]

Comparing self reported and physiological sleep quality from consumer devices to depression and neurocognitive performance

SUPPLEMENTARY MATERIALS

Supplementary Table 1. Absolute difference between week 6 and pre-scan passive and PSQI measurements per user.

Feature (PSQI or physiological)	Mean Abs. Difference	Standard Deviation	Min. Difference	Max. Difference	Count
Median Sleep Duration	0.63	0.63	0	4.44	153
Hours of Sleep - PSQI	0.75	0.93	0	5	154
Median Bedrest Onset	0.51	0.63	0	3.19	154
Bedtime - PSQI	3.48	7.15	0	23.75	154
Median Sleep Offset	0.66	0.72	0	4.11	153
Wakeup time - PSQI	1.06	1.87	0	13	154
Median Sleep Efficiency	0.07	0.07	0	0.57	153
Habitual Sleep Efficiency - PSQI	0.68	0.87	0	3	154
Median Sleep Onset Latency	0.49	0.81	0	6.81	153
Time to Fall Asleep (minutes) - PSQI	13.16	23.63	0	180	154
Awake Count	46.64	48.23	0	209	154
Wake up at night - PSQI	0.46	0.71	0	3	154

Supplementary Table 2. Model performance for predicting PHQ-14 item response with either self-reported sleep quality or physiological sleep quality reported as median sensitivity and specificity across cross validation folds. LR = Logistic Regression, RF = Random Forest, GB = Gradient Boosting, D = Dummy.

	PSQI (n=249 users, 705 responses)			Physiologic Sleep (n=247 users, 1565 responses)		
**PHQ-14 Item**	**Model**	**Sensitivity**	**Specificity**	**Model**	**Sensitivity**	**Specificity**
Trouble falling asleep or staying asleep	RF	0.972	0.452	GB	0.993	0.000
Feeling tired or having little energy	GB	1.000	0.000	LR	1.000	0.000
Little interest or pleasure in doing things	RF	1.000	0.000	RF	1.000	0.000
Sleeping too much	LR	0.800	0.612	RF	0.691	0.382
Feeling down, depressed	LR	1.000	0.000	RF	1.000	0.000
Feeling irritable	LR	1.000	0.000	D	1.000	0.000
Little interest in sex	LR	1.000	0.000	LR	0.991	0.000
Feeling hopeless	LR	0.981	0.099	GB	0.927	0.067
Trouble concentrating on things	RF	1.000	0.000	LR	1.000	0.000
Moving or speaking slowly	LR	0.068	0.983	GB	0.050	0.928
Feeling bad about yourself	LR	1.000	0.000	D	1.000	0.000
Poor appetite	LR	0.725	0.449	LR	0.742	0.242
Being fidgety or restless	LR	0.698	0.445	GB	0.554	0.485
Overeating	LR	0.831	0.204	D	1.000	0.000

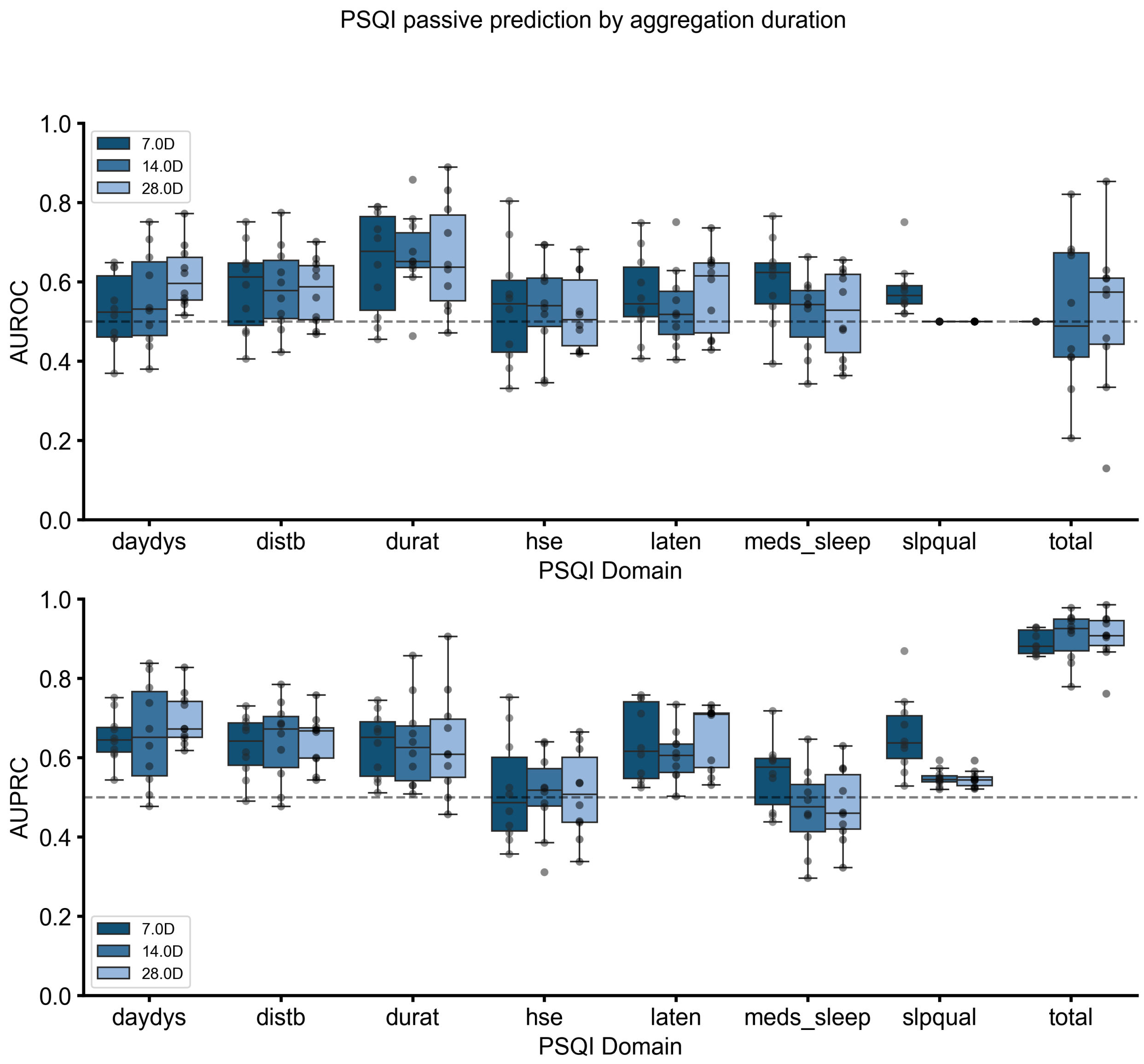

Supplementary Figure 1. Predicting self-reported sleep quality using physiological sleep over varying windows. Performance of predictive models at detecting domains and total scores of the Pittsburgh Sleep Quality Index (PSQI) with passive sensor data aggregated at different windows prior to self-report administration. Top plot shows AUROC score value and bottom plot shows average precision performance.

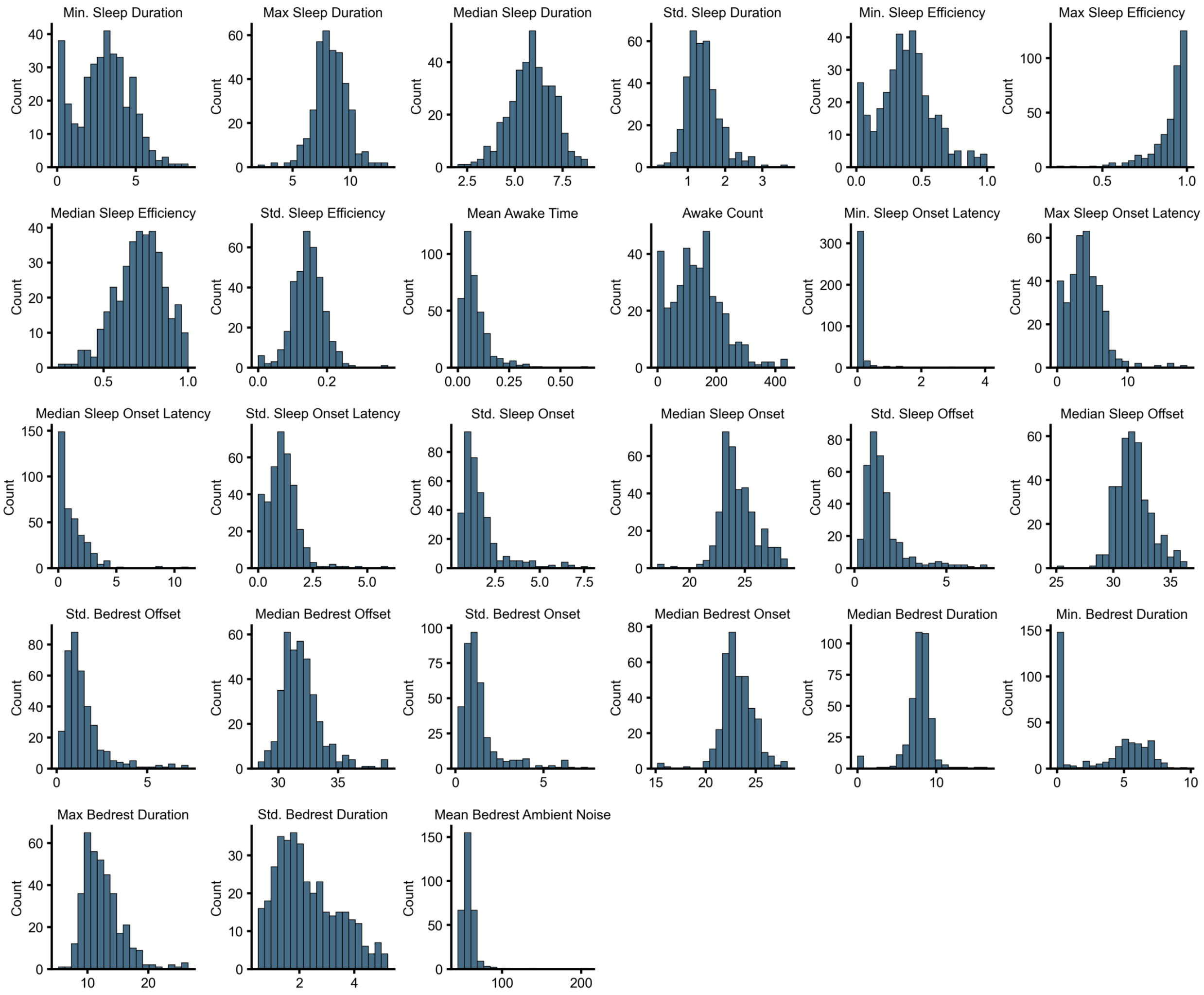

Supplementary Figure 2. Physiological Sleep Quality prior to PSQI. Histograms of passive sleep quality features aggregated 28-days prior to Pittsburgh Sleep Quality Index (PSQI) administrations.

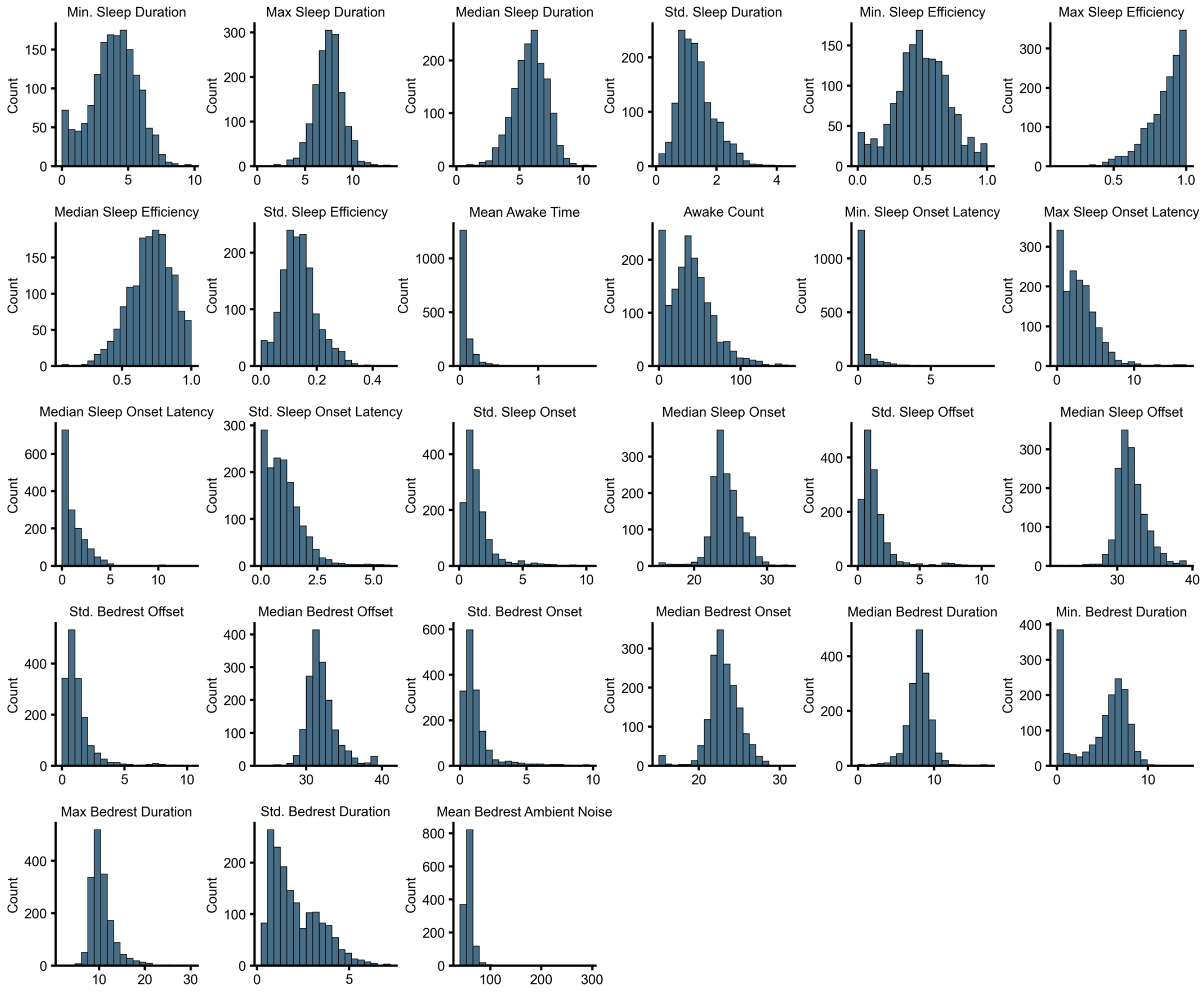

Supplementary Figure 3. Physiological Sleep Quality prior to PHQ-14. Histograms of passive sleep quality features aggregated 8-days prior to Patient Health Questionnaire-14 (PHQ-14) administrations.

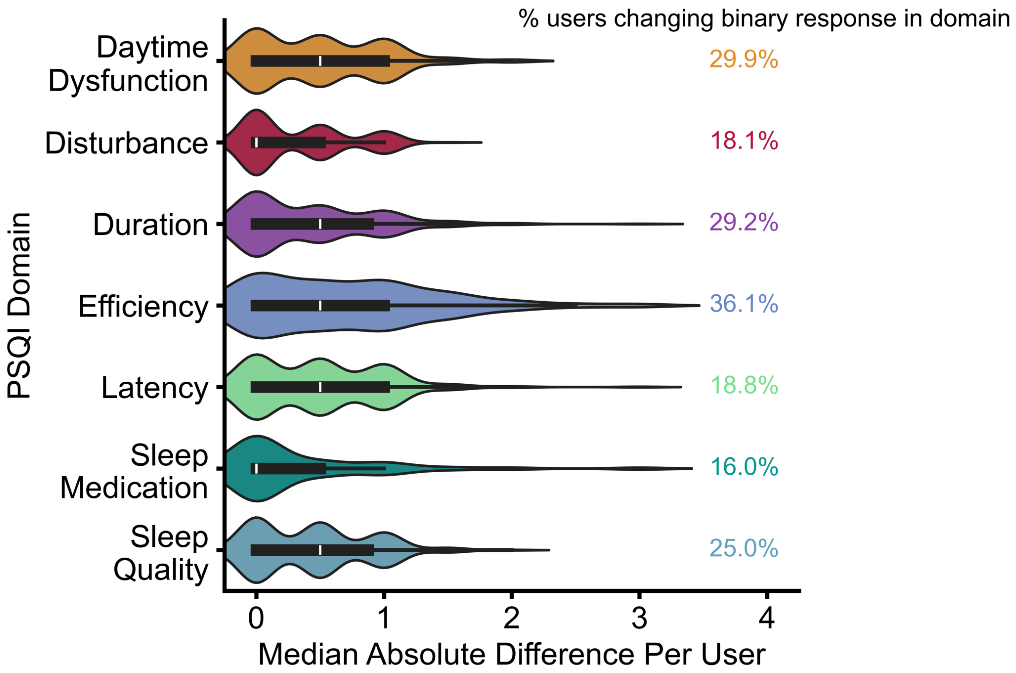

Supplementary Figure 4. Absolute difference between PSQI responses to each PSQI domain, median per user reported. Annotation on right side of plot refers to percentage of users who have a change in binarized response which is used as the predicted outcome.
